# Effect sizes of the differences between means without assuming variance equality and between a mean and a constant

**DOI:** 10.1016/j.heliyon.2020.e03306

**Published:** 2020-01-27

**Authors:** Satoshi Aoki

**Affiliations:** Graduate school of Science, the University of Tokyo, Bunkyo, Tokyo, Japan

**Keywords:** Psychology, Mathematics, Effect size, Standardized mean difference, Cohen's d, Hedges' d, Heteroscedasticity

## Abstract

Effect sizes of the difference, or standardized mean differences, are widely used for meta-analysis or power-analysis. However, common effect sizes of the difference such as Cohen's *d* or Hedges' *d* assume variance equality that is fragile and is often violated in practical applications. Based on Welch's *t* tests, we defined a new effect size of the difference between means, which did not assume variance equality, thereby providing a more accurate value for data with unequal variance. In addition, we presented the unbiased estimator of an effect size of the difference between a mean and a known constant. An R package is also provided to compute these effect sizes with their variance and confidence interval.

## 1 Introduction

Effect sizes of the difference or, more precisely, standardized mean differences between two groups, are widely used to estimate the magnitude of effect independent of the sample size [1], to conduct meta-analysis [2], or to conduct power-analysis [3]. The American Educational Research Association (AERA) or the American Psychological Association (APA) strongly recommend effect sizes are reported in the corresponding fields [4], [5]. Furthermore, the misuse and misunderstanding of p-value have become public [6], and use of effect sizes is spreading beyond pedagogy and psychology, where effect sizes have developed, into areas such as in biology [1]. In spite of such importance, the classical effect sizes of the difference assume variance equality (homoscedasticity), which is hard to assume practically or is even expected to be violated a priori in clinical data [7]. While Bonett [8] defined a confidence interval of an effect size estimator which did not assume homoscedasticity, its parameter was not defined. This problem of variance inequality (heteroscedasticity) has been long debated [9], [10]. In addition, the unbiased estimator of an effect size of the difference between a mean and a constant was undefined. To solve these problems, based on Welch's *t* test [11], [12], we defined an effect size of the difference between means that does not assume homoscedasticity and calculated the unbiased estimator of an effect size of the difference between a mean and a constant.

Effect size of the difference was developed by Cohen [13], who studied in the field of psychology. Cohen [3], [13] defined the effect size as a parameter for two independently and normally distributed populations, $Y^{1}∼N(\mu_{1},\sigma^{2})$ and $Y^{2}∼N(\mu_{2},\sigma^{2})$:

(1) $$\delta =(\mu_{1}-\mu_{2})/\sigma ,$$

which is expressed as *d* in the original articles [3], [13]. Note that both populations share the common variance $\sigma^{2}$. The estimator of this parameter was represented as $d_{s}$ in [3]. However, we refer to this estimating statistic as *g* to distinguish it from the other *d* we introduce later. The statistic *g* is defined as

(2) $$g=({\overset{¯}{Y}}^{1}-{\overset{¯}{Y}}^{2})/S^{\text{pooled}},$$

where

$$S^{\text{pooled}}=\sqrt{\frac{s_{1}^{2}(n_{1}-1)+s_{2}^{2}(n_{2}-1)}{n_{1}+n_{2}-2}},$$

and, for i=1,2,

(3) $$s_{i}^{2}=\frac{\sum_{j=1}^{n_{i}}{\left(Y_{j}^{i}-\overset{¯}{Y^{i}}\right)}^{2}}{n_{i}-1}.$$

Here, ${\overset{¯}{Y}}^{1}$, $Y_{j}^{1}$, and $n_{1}$ are the mean of the sample, the sample (random variable), and the sample size of group 1, respectively, while ${\overset{¯}{Y}}^{2}$, $Y_{j}^{2}$, and $n_{2}$ are those of group 2. For the denominator, this effect size uses the pooled standard deviation, which suggests the most precise population variance under the assumption of equal variance [14].

In the field of pedagogy, Glass [2] suggested another effect size of the difference, independently of Cohen's works. He defined it as “the mean difference on the outcome variable between treated and untreated subjects divided by the within group standard deviation,” where “the within group standard deviation” corresponds to the standard deviation of the untreated group. He clearly distinguished the treated (experimental) group from the untreated (control) group, and there was no assumption regarding the two groups. His effect size was subsequently formulated and named Glass' Δ by Hedges [14], which is

(4) $$\Delta =({\overset{¯}{Y}}^{E}-{\overset{¯}{Y}}^{C})/S^{C},$$

where $\overset{¯}{Y^{E}}$ is the mean of the variable in the experimental group, $\overset{¯}{Y^{C}}$ is that in the control group, and $S^{C}$ is the unbiased standard deviation of the control group.

Hedges [14] also defined the *δ* (1) and the *g* (2) independently of Cohen. Furthermore, Hedges [14] indicated that *g* (2) is biased from *δ* (1), making it unsuitable for analyses that do not treat the entire population. The unbiased estimator of *δ* (1) is defined as $g^{U}$ in [14] and *d* in [15]. In this study, we call it *d*, which is

(5) $$d=J(n_{1}+n_{2}-2)g.$$

Using the gamma function, the correction coefficient *J* is defined as

(6) $$J(m)=\frac{\Gamma (m/2)}{\sqrt{m/2}\Gamma \{(m-1)/2\}}.$$

The effect sizes *g* (2) and *d* (5) are used in various fields of science, but they assume homoscedasticity just like Student's t-test [16], [17]. When this assumption of homoscedasticity is violated, Grissom [9] recommended the use of Glass's Δ (4) instead of *d* (5). However, Glass's Δ (4) and *d* (5) have different meaning because of the difference in denominator. Therefore, Glass's Δ (4) cannot substitute for *d* (5) in a strict sense. Behavior of *g* (2), Δ (4), and *d* (5) under heteroscedasticity was studied in [10], although the justification for using effect size parameter *α*, that they defined, to measure the statistic bias under heteroscedasticity was not shown.

Bonett [8] in psychology proposed a confidence interval (CI) of effect size which does not assume homoscedasticity. First, he defined a general effect size estimator

(7) $$\overset{ˆ}{\delta }=\sum_{j=1}^{k}c_{j}{\overset{¯}{Y}}_{j}/s,$$

where $\sum_{j=1}^{k}c_{j}=0$, ${\overset{¯}{Y}}_{j}$ is a sample mean, and $s=\sqrt{k^{-1}\sum_{j=1}^{k}s_{j}^{2}}$. Concerning effect size of the difference between two means, substituting k=2, $c_{1}=1$ and $c_{2}=-1$ gives

(8) $$\overset{ˆ}{\delta }=\frac{{\overset{¯}{Y}}^{1}-{\overset{¯}{Y}}^{2}}{\sqrt{(s_{1}^{2}+s_{2}^{2})/2}}.$$

Then, he assumed its corresponding parameter and its CI. The CI was calculated using approximation of CI [18] and variance of the estimator which was approximately calculated without assuming homoscedasticity. The parameters estimated by $\overset{ˆ}{\delta }$ (7) or (8) were not formulated. Namely, he defined the CI for heteroscedasticity without defining a parameter, and this can be a problem. When the estimator does not always correspond to a single parameter, the CI of an undefined parameter loses its consistency in what to estimate, and heteroscedasticity or difference of sample sizes can change the correspondence between an estimator and a parameter (see section 5.2). Although his CI was effective relative to the other CIs in his simulation experiment where the parameter was given a value, what the value meant could change depending on the variance and sample size, and the change could not be expected since the parameter was not formulated.

It should be noted, Cohen [3] also defined a parameter of an effect size of the difference between a mean and a constant for a normally distributed population $N_{1}(\mu ,\sigma_{1}^{2})$ and a known constant *C* as

(9) $$\gamma =(\mu -C)/\sigma_{1}.$$

Cohen [3] originally referred to this as $d_{3}^{\prime }$, but we refer to this as *γ* (9) to clearly distinguish it from *d* (5). Cohen [3] also defined a biased estimator of an effect size for a normally distributed population with the sample value $Y_{i}^{1}$ ($i=1,...,n_{1}$), the sample mean $\overset{¯}{Y^{1}}$, and a known constant *C* as

(10) $$c^{\text{biased}}=({\overset{¯}{Y}}^{1}-C)/s_{1}.$$

The $s_{1}$ is the square root of (3). Cohen [3] originally referred to this as $d_{s}^{\prime }$, but we refer this to $c^{\text{biased}}$ for the reason described above. To the best of my knowledge, the unbiased estimator of *γ* (9) has not been shown.

There are other effect sizes of the difference that do not assume normality or independence. Since their assumption is different from that of effect size we focus on, we do not treat them in detail and briefly introduce them. Dunlap et al. [19] invented effect size of the difference between two correlated paired groups. Algina et al. [20] proposed robust effect size of the difference, which is based on *g* (2) using 20% trimmed mean and 20% Winsorized variance assuming that samples are taken from an observing population and another contaminating population.

## 2 Theory

### 2.1 An effect size of the difference between means without assuming homoscedasticity

First, we define the parameter of an effect size of the difference between means for two independently and normally distributed populations $N_{1}(\mu_{1},\sigma_{1}^{2})$ and $N_{2}(\mu_{2},\sigma_{2}^{2})$ as

(11) $$\epsilon_{r}=\frac{\mu_{1}-\mu_{2}}{\sqrt{\left(\sigma_{1}^{2}+r\sigma_{2}^{2})/(r+1\right)}},$$

where *r* is a non-negative real number. This parameter is not generalization of *δ* (1) and is different from it. Then, suppose two independently and normally distributed populations with the samples $Y_{i}^{1}$ ($i=1,...,n_{1}$) and $Y_{i}^{2}$ ($i=1,...,n_{2}$), and the sample mean ${\overset{¯}{Y}}^{1}$ and ${\overset{¯}{Y}}^{2}$. Based on the statistic $t_{w}$, the so-called Welch's *t* [11], [12], a biased estimator of $\epsilon_{r}$ (11) is defined as

(12) $$e^{\text{biased}}=t_{w}/\sqrt{\overset{˜}{n}},$$

where

(13) $$t_{w}=\frac{{\overset{¯}{Y}}^{1}-{\overset{¯}{Y}}^{2}}{\sqrt{s_{1}^{2}/n_{1}+s_{2}^{2}/n_{2}}},$$

$s_{i}^{2}$ is the same as (3), and

(14) $$\overset{˜}{n}=n_{1}n_{2}/(n_{1}+n_{2}).$$

Finally, *e*, the unbiased estimator of $\epsilon_{r}$ (11), is

(15) $$e=e^{\text{biased}}J(f).$$

Therefore,

$$E(e)=\epsilon_{r}.$$

Here, *r* corresponds to the ratio $n_{1}/n_{2}$. *J* is the correction coefficient that is defined in equation (6). The degree of freedom *f* is approximately calculated using the Welch-Satterthwaite equation [11], [21] as

(16) $$f=\frac{{\left(s_{1}^{2}/n_{1}+s_{2}^{2}/n_{2}\right)}^{2}}{s_{1}^{4}/\{n_{1}^{2}(n_{1}-1)\}+s_{2}^{4}/\{n_{2}^{2}(n_{2}-1)\}}.$$

The variance of *e* (15) is

$$\mathrm{var}(e)=\frac{f}{f-2}J^{2}(f)(1/\overset{˜}{n}+\epsilon_{r}^{2})-\epsilon_{r}^{2}.$$

Although this effect size is derived from the difference, we refer to it as *e* not *d*. This is because Cohen's *d* (2) and Hedges' *d* (5) already exist, and more *d* would cause further confusion. The proof of the bias correction and variance derivation does not assume homoscedasticity (see the Appendix). In addition, *e* (15) is a consistent estimator of $\epsilon_{r}$ (11) at the same time. See the Appendix for the proof of the consistency.

### 2.2 An effect size of the difference between a mean and a known constant

Using $c^{\text{biased}}$ (10), the unbiased estimator of the effect size parameter *γ* (9) is defined for a normally distributed population with the sample value $Y_{i}^{1}$ ($i=1,...,n_{1}$), the sample mean $\overset{¯}{Y^{1}}$, and a known constant *C* as

(17) $$c=c^{\text{biased}}J(n_{1}-1).$$

Therefore,

E(c)=γ.

The correction coefficient *J* (6) is the same as the one used above. The variance of *c* is

$$\mathrm{var}(c)=\frac{n_{1}-1}{n_{1}-3}J^{2}(n_{1}-1)(\frac{1}{n_{1}-1}+\gamma^{2})-\gamma^{2}.$$

See the Appendix for proofs of the bias correction and the derivation of the variance. In addition, *c* (17) is a consistent estimator of *γ* (9) (see the Appendix for the proof). When interested in constants rather than variables, $c^{\prime }$ defined as

$$c^{\prime }=(C-{\overset{¯}{Y}}^{1})J(n_{1}-1)/s_{1}$$

can be used instead of *c*.

### 2.3 Confidence intervals of effect sizes

In terms of the effect sizes of the difference, the CI based on a noncentral t variate is not directly given by a formula [22]. The CI is derived from that of noncentral parameters of noncentral t-distribution, which is in turn obtained by some searching method. The CI based on the biased effect sizes are given as:

$$[ncp_{L}/\sqrt{\overset{˜}{n}},\;\;ncp_{H}/\sqrt{\overset{˜}{n}}]:\;\;CI\;\;based\;\;on\;\;g\;\;and\;\;e^{\text{biased}},$$

and

$$[ncp_{L}/\sqrt{n_{1}-1},\;\;ncp_{H}/\sqrt{n_{1}-1}]:\;\;CI\;\;based\;\;on\;\;c^{\text{biased}},$$

where $ncp_{L}$ is the noncentral parameter that gives the upper limit of cumulative probability (e.g., 0.975 cumulative probability for 95% CI) for noncentral t-distribution with the corresponding t value (see the discussion section) and the degree of freedom, and $ncp_{H}$ is that which gives the lower limit (e.g., 0.025 cumulative probability for 95% CI), and $\overset{˜}{n}$ and $n_{1}$ are the same as (14) and (10). The CIs based on the unbiased estimator of the effect sizes are given by multiplying the corresponding correction coefficient *J* (6) of the corresponding degree of freedom to the above intervals.

The CI by Bonett [8] is calculated using variance of the estimator which is approximately calculated without assuming homoscedasticity and approximate assumption of CI [18]. Therefore, it is not necessary to apply Bonett's CI to *e* (15) or *c* (17), because the derivation of their CIs does not assume homoscedasticity, and their exact CIs can be calculated without approximation.

## 3 Calculation method

I developed a new package es.dif for R [23]. It enables the statistics *d* (5), *e* (15), *c* (17), their biased statistics, variance, and CI based on the two samples or their mean, variance, and sample size to be computed. In this package, approximation of *J* (6) [14] is not employed unless its degree of freedom exceeds 342, when the gamma function returns values that are too large to be treated in R. The CI is obtained by binary search.

The remainder of this section presents some examples of the package. First, the following script calculates d (5), e (15), their variances and 95% CIs for data 1 (0,1,2,3,4) and data 2 (0,0,1,2,2).

> library(es.dif)  > data1<-c(0,1,2,3,4)  > data2<-c(0,0,1,2,2)  > es.d(data1,data2)   [,1] [,2]  [1,] "Hedges' d:" "0.682379579593354"  [2,] "variance:" "0.484026380702367"  [3,] "CI:" "[ -0.503527216375147 ,  1.82938058482178 ]"  > es.e(data1,data2)   [,1] [,2]  [1,] "Unbiased e:" "0.668264936033828"  [2,] "variance:" "0.506830833214916"  [3,] "CI:" "[ -0.50334965496395 ,  1.7965317007171 ]"

Using options of the function, you can change the type I error rate for the CI, calculate biased effect sizes, and output results in the vector style. For example, $c^{\text{biased}}$ (10) with 99% CI in the vector style is calculated by this script.

> library(es.dif)  > data1<-c(0,0,1,2,2)  > data2<-c(2)  > es.c(data1,data2,alpha=0.01,  unbiased=FALSE,vector_out=TRUE)  [1] -1.0000000 0.9292037 -2.5390625 0.5778885

In the vector-style output, the four values in the vector show the effect size, its variance, and lower and higher limits of the CI. In addition, this package includes functions that can output effect sizes from the (estimated) parameters and the sample sizes. The following scripts compute *d* (5) and *e* (15) for two populations, N(1,2) and N(0,1) with the sample size 5 and 10, respectively.

> library(es.dif)  > mean1<-1  > mean2<-0  > var1<-2  > var2<-1  > n1<-5  > n2<-10  > es.para.d(mean1,mean2,var1,var2,n1,n2)   [,1] [,2]  [1,] "Hedges' d:" "0.82286529714397"  [2,] "variance:" "0.349443397657368"  [3,] "CI:" "[ -0.248827687382689 ,  1.86616833367494 ]"  > es.para.e(mean1,mean2,var1,var2,n1,n2)   [,1] [,2]  [1,] "Unbiased e:" "0.674259756444758"  [2,] "variance:" "0.41613476136966"  [3,] "CI:" "[ -0.354146439977423 ,  1.65626025590509 ]"

These types of functions also have the options for the type I error rate, the biased effect size, and the vector-style output.

## 4 Application & simulation

While the situation to use *c* (17) is clearly different, the *e* (15) and *d* (5) have a similar application range in practice. Therefore, we prepared an example of the applications in which the sample variances are not equal. Table 1 shows well-known data of three *Iris* species by Fisher [24], which can also be checked in R [23] using a command “iris”. Note that only the petal width of *I. setosa* has fewer significant digits. For this data, we calculated *d* (5), *e* (15), the ratio of *d* (5) to *e* (15), and the ratio of the standard deviations of the two comparing data. Theoretically, *e* (15) is a more precise estimator of its own parameter than *d* (5) under this heteroscedasticity.

**Table 1:** Measured characteristics (in centimeters) of three Iris species shown in Fisher (1936).

*Iris setosa*				*Iris versicolor*				*Iris virginica*			
S.L.	S.W.	P.L.	P.W.	S.L.	S.W.	P.L.	P.W.	S.L.	S.W.	P.L.	P.W.
5.1	3.5	1.4	0.2	7.0	3.2	4.7	1.4	6.3	3.3	6.0	2.5
4.9	3.0	1.4	0.2	6.4	3.2	4.5	1.5	5.8	2.7	5.1	1.9
4.7	3.2	1.3	0.2	6.9	3.1	4.9	1.5	7.1	3.0	5.9	2.1
4.6	3.1	1.5	0.2	5.5	2.3	4.0	1.3	6.3	2.9	5.6	1.8
5.0	3.6	1.4	0.2	6.5	2.8	4.6	1.5	6.5	3.0	5.8	2.2
5.4	3.9	1.7	0.4	5.7	2.8	4.5	1.3	7.6	3.0	6.6	2.1
4.6	3.4	1.4	0.3	6.3	3.3	4.7	1.6	4.9	2.5	4.5	1.7
5.0	3.4	1.5	0.2	4.9	2.4	3.3	1.0	7.3	2.9	6.3	1.8
4.4	2.9	1.4	0.2	6.6	2.9	4.6	1.3	6.7	2.5	5.8	1.8
4.9	3.1	1.5	0.1	5.2	2.7	3.9	1.4	7.2	3.6	6.1	2.5
5.4	3.7	1.5	0.2	5.0	2.0	3.5	1.0	6.5	3.2	5.1	2.0
4.8	3.4	1.6	0.2	5.9	3.0	4.2	1.5	6.4	2.7	5.3	1.9
4.8	3.0	1.4	0.1	6.0	2.2	4.0	1.0	6.8	3.0	5.5	2.1
4.3	3.0	1.1	0.1	6.1	2.9	4.7	1.4	5.7	2.5	5.0	2.0
5.8	4.0	1.2	0.2	5.6	2.9	3.6	1.3	5.8	2.8	5.1	2.4
5.7	4.4	1.5	0.4	6.7	3.1	4.4	1.4	6.4	3.2	5.3	2.3
5.4	3.9	1.3	0.4	5.6	3.0	4.5	1.5	6.5	3.0	5.5	1.8
5.1	3.5	1.4	0.3	5.8	2.7	4.1	1.0	7.7	3.8	6.7	2.2
5.7	3.8	1.7	0.3	6.2	2.2	4.5	1.5	7.7	2.6	6.9	2.3
5.1	3.8	1.5	0.3	5.6	2.5	3.9	1.1	6.0	2.2	5.0	1.5
5.4	3.4	1.7	0.2	5.9	3.2	4.8	1.8	6.9	3.2	5.7	2.3
5.1	3.7	1.5	0.4	6.1	2.8	4.0	1.3	5.6	2.8	4.9	2.0
4.6	3.6	1.0	0.2	6.3	2.5	4.9	1.5	7.7	2.8	6.7	2.0
5.1	3.3	1.7	0.5	6.1	2.8	4.7	1.2	6.3	2.7	4.9	1.8
4.8	3.4	1.9	0.2	6.4	2.9	4.3	1.3	6.7	3.3	5.7	2.1
5.0	3.0	1.6	0.2	6.6	3.0	4.4	1.4	7.2	3.2	6.0	1.8
5.0	3.4	1.6	0.4	6.8	2.8	4.8	1.4	6.2	2.8	4.8	1.8
5.2	3.5	1.5	0.2	6.7	3.0	5.0	1.7	6.1	3.0	4.9	1.8
5.2	3.4	1.4	0.2	6.0	2.9	4.5	1.5	6.4	2.8	5.6	2.1
4.7	3.2	1.6	0.2	5.7	2.6	3.5	1.0	7.2	3.0	5.8	1.6
4.8	3.1	1.6	0.2	5.5	2.4	3.8	1.1	7.4	2.8	6.1	1.9
5.4	3.4	1.5	0.4	5.5	2.4	3.7	1.0	7.9	3.8	6.4	2.0
5.2	4.1	1.5	0.1	5.8	2.7	3.9	1.2	6.4	2.8	5.6	2.2
5.5	4.2	1.4	0.2	6.0	2.7	5.1	1.6	6.3	2.8	5.1	1.5
4.9	3.1	1.5	0.1	5.4	3.0	4.5	1.5	6.1	2.6	5.6	1.4
5.0	3.2	1.2	0.2	6.0	3.4	4.5	1.6	7.7	3.0	6.1	2.3
5.5	3.5	1.3	0.2	6.7	3.1	4.7	1.5	6.3	3.4	5.6	2.4
4.9	3.1	1.5	0.1	6.3	2.3	4.4	1.3	6.4	3.1	5.5	1.8
4.4	3.0	1.3	0.2	5.6	3.0	4.1	1.3	6.0	3.0	4.8	1.8
5.1	3.4	1.5	0.2	5.5	2.5	4.0	1.3	6.9	3.1	5.4	2.1
5.0	3.5	1.3	0.3	5.5	2.6	4.4	1.2	6.7	3.1	5.6	2.4
4.5	2.3	1.3	0.3	6.1	3.0	4.6	1.4	6.9	3.1	5.1	2.3
4.4	3.2	1.3	0.2	5.8	2.6	4.0	1.2	5.8	2.7	5.1	1.9
5.0	3.5	1.6	0.6	5.0	2.3	3.3	1.0	6.8	3.2	5.9	2.3
5.1	3.8	1.9	0.4	5.6	2.7	4.2	1.3	6.7	3.3	5.7	2.5
4.8	3.0	1.4	0.3	5.7	3.0	4.2	1.2	6.7	3.0	5.2	2.3
5.1	3.8	1.6	0.2	5.7	2.9	4.2	1.3	6.3	2.5	5.0	1.9
4.6	3.2	1.4	0.2	6.2	2.9	4.3	1.3	6.5	3.0	5.2	2.0
5.3	3.7	1.5	0.2	5.1	2.5	3.0	1.1	6.2	3.4	5.4	2.3
5.0	3.3	1.4	0.2	5.7	2.8	4.1	1.3	5.9	3.0	5.1	1.8
5.0	3.4	1.5	0.2	5.9	2.8	4.3	1.3	6.6	3.0	5.6	2.0
0.35	0.38	0.17	0.1	0.52	0.31	0.47	0.20	0.64	0.32	0.55	0.27

*Note:* S.L. = sepal length; S.W. = sepal width; P.L. = petal length; P.W. = petal width. The last two rows show the average and the standard deviation of the corresponding column.

The calculated result is shown in Table 2. When considering their significant digits, the comparing pair of the sepal length of *I. setosa* and *I. virginica* showed the different effect size of *d* (5) and *e* (15) (in bold in Table 2). Even though most pairs showed identical values of *d* (5) and *e* (15), the result revealed that violation of the assumption of homoscedasticity in *d* (5) can affect the result even in two significant digits.

**Table 2:** Calculated effect sizes of the difference for the data shown in Table 1.

Chara.	Taxa	d	e	d/e	sd ratio
S.L.	1 vs 2	-2.1	-2.1	1.001029	0.682893
	1 vs 3	**-3.1**	**-3.0**	1.002185	0.554334
	2 vs 3	-1.1	-1.1	1.000328	0.811744
S.W.	1 vs 2	1.8	1.8	1.000285	1.214233
	1 vs 3	1.2	1.2	1.000212	1.181483
	2 vs 3	-0.64	-0.64	1.000006	0.973028
P.L.	1 vs 2	-7.8	-7.8	1.004510	0.369243
	1 vs 3	-9.9	-9.9	1.005256	0.314392
	2 vs 3	-2.5	-2.5	1.000197	0.851450
P.W.	1 vs 2	-7	-7	1.002318	0.542139
	1 vs 3	-8	-8	1.004222	0.390349
	2 vs 3	-2.9	-2.9	1.000781	0.720017

*Note:* Chara. = characteristics; S.L. = sepal length; S.W. = sepal width; P.L. = petal length; P.W. = petal width; Taxa = compared taxa; 1 = *I. setosa*; 2 = *I. versicolor*; 3 = *I. virginica*; d = effect size *d*(5); e = effect size *e*(15). These effect sizes are shown in the original significant digits. d/e = the ratio of *d*(5) to *e*(15); sd ratio = the ratio of the standard deviations of the compared data. Note that reverse comparisons, such as 2 vs 1, were also conducted, but omitted from this table because their effect sizes are the opposites of the original values, and d/e and sd ratio are the inverses of the original ones.

**Figure 1:**
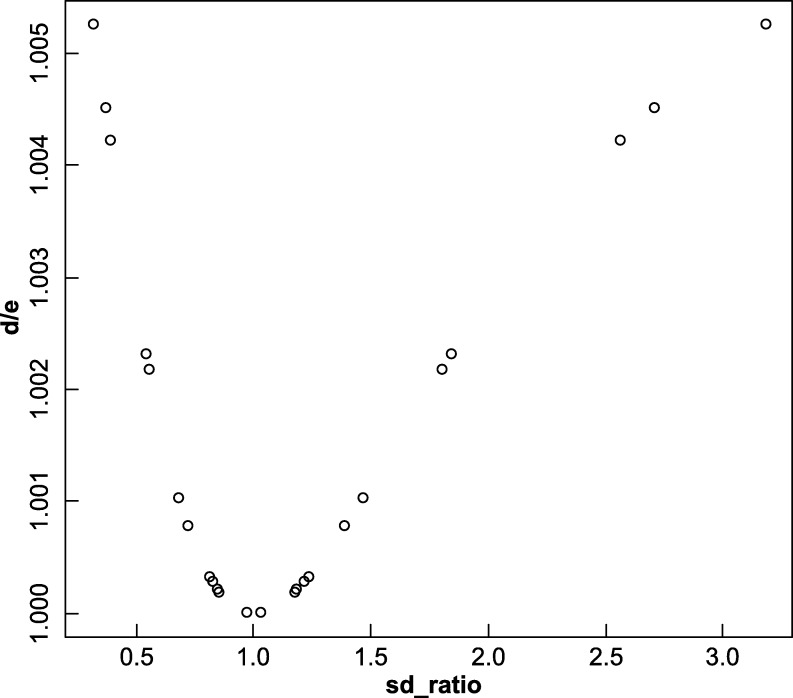
Plotted graph of Table 2.

Fig. 1 shows the ratio of *d* (5) to *e* (15) plotted against the ratio of standard deviations of the comparing data. This figure shows that the similar two standard deviations give similar *d* (5) and *e* (15). In other words, the more different two standard deviations encourage the use of *e* (15) over *d* (5) more.

To examine the nature of *d* (5) and *e* (15), we also conducted a simulation study. In addition to *d* (5) and *e* (15), Bonett's statistic $\overset{ˆ}{\delta }$ (8) was also included as a reference, although its accuracy cannot be discussed because of the lack of the parameter definition. The above effect sizes and their width of 95% CI were calculated for 100,000 Monte Calro replications from $N(1,\sigma_{1}^{2})$ and $N(0,\sigma_{2}^{2})$ for each condition, and they were represented by their average values. The population means were fixed to 1 and 0. The sample sizes were changed from 10 to 30 by 10. The population standard deviation $\sigma_{1}$ was fixed to 1 and $\sigma_{2}$ was changed 1 to 10 by 1. However, some redundant data were omitted from the result. The calculation was conducted using es.dif R package shown above and metafor R package [25]. The R source code used for the simulation was shown in the Appendix.

Table 3 shows the result of the simulation. When the sample size ratio was conserved under $\sigma_{1}\neq \sigma_{2}$, *e* (15) gave more similar and concordant values than *d* (5). For example, *e* (15) for $n_{1}=n_{2}=10,20,30$ under $\sigma_{2}=10$ were 0.142, 0.140 and 0.141, whereas the corresponding *d* (5) were 0.148, 0.143 and 0.143. This is the nature and advantage of *e* (15) which is designed to estimate the same parameter under heteroscedasticity and the same sample size ratio. The width of CI was narrowest for *d* (5) under $\sigma_{1}=\sigma_{2}$, and *e* (15) had the second narrowest. Under $\sigma_{1}\neq \sigma_{2}$, *e* (15), *e* (15) and $\overset{ˆ}{\delta }$ (8) had the narrowest CI under $n_{1}=n_{2}$, $n_{1}>n_{2}$ and $n_{1}<n_{2}$, respectively. The narrowest CIs of *e* (15) were followed by *d* (5), whereas what followed the narrowest CIs of $\overset{ˆ}{\delta }$ (8) was not fixed. It was shown that *e* (15) had wider situation under which it had the narrowest or second narrowest CI than *d* (5) or $\overset{ˆ}{\delta }$ (8). Bonett's statistic $\overset{ˆ}{\delta }$ (8) equaled to *d* (5) under $n_{1}=n_{2}$ as their definition. Under $n_{1}\neq n_{2}$ and $\sigma_{1}\neq \sigma_{2}$, *e* (15) was closer to $\overset{ˆ}{\delta }$ (8) than *d* (5). This might imply relative accuracy of $\overset{ˆ}{\delta }$ (8) over *d* (5) under heteroscedasticity.

**Table 3:** Comparison of effect sizes in simulation.

*n*_1_	*n*_2_	*σ*_1_	*σ*_2_	d.ES	d.Par.	e.ES	e.Par.	B.ES	B.Par.	d.CI	e.CI	B.CI
10	10	1	1	1.000	1.000	0.995	1.000	1.000	U.D.	1.823	1.828	1.911
10	10	1	4	0.355	N.C.	0.344	0.343	0.355	U.D.	1.722	1.691	1.834
10	10	1	7	0.210	N.C.	0.201	0.200	0.210	U.D.	1.713	1.668	1.825
10	10	1	10	0.148	N.C.	0.142	0.141	0.148	U.D.	1.710	1.661	1.822
10	20	1	1	0.998	1.000	0.997	1.000	1.002	U.D.	1.579	1.604	1.646
10	20	1	4	0.303	N.C.	0.408	0.408	0.346	U.D.	1.493	1.503	1.310
10	20	1	7	0.175	N.C.	0.243	0.243	0.203	U.D.	1.487	1.488	1.274
10	20	1	10	0.124	N.C.	0.173	0.171	0.143	U.D.	1.486	1.483	1.264
10	30	1	1	0.999	1.000	1.000	1.000	1.007	U.D.	1.482	1.536	1.551
10	30	1	4	0.284	N.C.	0.458	0.459	0.343	U.D.	1.412	1.427	1.096
10	30	1	7	0.164	N.C.	0.278	0.277	0.202	U.D.	1.408	1.416	1.046
10	30	1	10	0.114	N.C.	0.196	0.197	0.141	U.D.	1.407	1.411	1.032
20	10	1	1	1.002	1.000	1.001	1.000	1.006	U.D.	1.580	1.605	1.647
20	10	1	4	0.431	N.C.	0.302	0.302	0.360	U.D.	1.523	1.462	1.830
20	10	1	7	0.262	N.C.	0.175	0.174	0.213	U.D.	1.515	1.443	1.843
20	10	1	10	0.186	N.C.	0.123	0.122	0.151	U.D.	1.512	1.438	1.846
20	20	1	1	0.999	1.000	0.998	1.000	0.999	U.D.	1.303	1.305	1.333
20	20	1	4	0.347	N.C.	0.342	0.343	0.347	U.D.	1.234	1.227	1.277
20	20	1	7	0.204	N.C.	0.200	0.200	0.204	U.D.	1.227	1.214	1.268
20	20	1	10	0.143	N.C.	0.140	0.141	0.143	U.D.	1.225	1.211	1.266
20	30	1	1	1.001	1.000	1.000	1.000	1.001	U.D.	1.189	1.199	1.215
20	30	1	4	0.318	N.C.	0.379	0.378	0.346	U.D.	1.125	1.129	1.050
20	30	1	7	0.184	N.C.	0.222	0.222	0.201	U.D.	1.120	1.119	1.032
20	30	1	10	0.130	N.C.	0.157	0.157	0.142	U.D.	1.119	1.115	1.027
30	10	1	1	0.998	1.000	0.999	1.000	1.005	U.D.	1.482	1.536	1.551
30	10	1	4	0.486	N.C.	0.285	0.286	0.362	U.D.	1.447	1.378	1.833
30	10	1	7	0.304	N.C.	0.166	0.164	0.216	U.D.	1.442	1.360	1.854
30	10	1	10	0.212	N.C.	0.113	0.115	0.148	U.D.	1.440	1.355	1.859
30	20	1	1	0.999	1.000	0.999	1.000	1.000	U.D.	1.189	1.199	1.215
30	20	1	4	0.386	N.C.	0.316	0.316	0.349	U.D.	1.133	1.120	1.268
30	20	1	7	0.229	N.C.	0.184	0.183	0.205	U.D.	1.127	1.108	1.269
30	20	1	10	0.161	N.C.	0.129	0.129	0.144	U.D.	1.125	1.105	1.269
30	30	1	1	1.002	1.000	1.001	1.000	1.002	U.D.	1.067	1.069	1.084
30	30	1	4	0.347	N.C.	0.343	0.343	0.347	U.D.	1.011	1.010	1.037
30	30	1	7	0.204	N.C.	0.202	0.200	0.204	U.D.	1.006	1.000	1.029
30	30	1	10	0.143	N.C.	0.141	0.141	0.143	U.D.	1.005	0.998	1.027

*Note:* d = effect size *d*(5); e = effect size *e*(15); B = effect size ${\overset{ˆ}{\delta }}^{\prime }$(8); Par. = parameter of effect size; CI = width of confidence interval; N.C. = not calculable; U.D. = undefined. The narrowest CI in each row is underlined.

## 5 Discussion

### 5.1 Correspondence of effect sizes and *t* tests

Comparison of *t* tests and the effect sizes of the difference except $\overset{ˆ}{\delta }$ (8) shows the clear correspondence between them (Table 4). Statistic *d* (5) corresponds to the unpaired two-sample *t* test [16], [17], whose statistic is the basis of *g* (2). Statistic $e^{\text{biased}}$ (12) uses the statistic (13) of Welch's *t* test [12], which aims to test two means with unequal variances, and $c^{\text{biased}}$ (10) uses the same statistic as the one-sample *t* test [17]. Considering this, it is natural that power analyses should be conducted, using the corresponding pair of the effect size and *t* test. In other words, power analyses of Student's one-sample *t* test, Student's unpaired two-sample *t* test, and Welch's *t* test should be conducted based on the *c* statistic (17), *d* (5), and the *e* statistic (15), respectively. Co-use of non-corresponding t-test and effect size causes inconsistence of the assumption about the population(s).

**Table 4:** Correspondence of assumptions, t values, and effect sizes of the difference.

	One sample & a constant	Two samples under homoscedasticity	Two samples under heteroscedasticity
As.	Normality	Normality, Independence, & Homoscedasticity	Normality & Independence
t	$t=\frac{\overset{¯}{Y^{1}}-C}{\sqrt{s_{1}^{2}/(n_{1}-1)}}$	$t=\frac{\overset{¯}{Y^{1}}-\overset{¯}{Y^{2}}}{S^{\text{pooled}}/\sqrt{\overset{˜}{n}}}$	$t=\frac{\overset{¯}{Y^{1}}-\overset{¯}{Y^{2}}}{\sqrt{s_{1}^{2}/n_{1}+s_{2}^{2}/n_{2}}}$
ES	$c=\frac{\overset{¯}{Y^{1}}-C}{s_{1}}J$	$d=\frac{\overset{¯}{Y^{1}}-\overset{¯}{Y^{2}}}{S^{\text{pooled}}}J$	$e=\frac{\overset{¯}{Y^{1}}-\overset{¯}{Y^{2}}}{\sqrt{(s_{1}^{2}/n_{1}+s_{2}^{2}/n_{2})\overset{˜}{n}}}J$

*Note:* As. = assumption; t = t value; ES = effect size. The degree of freedom of J is omitted for the space and must be calculated corresponding degree of freedom.

### 5.2 Influence of sample size on effect size

In this subsection, the relationship between the effect sizes of the difference and sample sizes is described. The value of *g* (2), a biased estimator of the effect size of the difference under homoscedasticity, is independent of the sample sizes when the assumption of homoscedasticity ($s_{1}=s_{2}$) is fulfilled. When $s_{1}\neq s_{2}$, it depends on the ratio $q=(n_{1}-1)/(n_{2}-1)$ as implied in [9]. This is because *g* (2) is no longer an estimator of *δ* (1) under $s_{1}\neq s_{2}$, and it will be a biased estimator of the other parameter $\delta_{q}^{\prime }$, which is

$$\delta_{q}^{\prime }=\frac{\mu_{1}-\mu_{2}}{\sqrt{\left(q\sigma_{1}^{2}+\sigma_{2}^{2})/(1+q\right)}}.$$

Note that even *d* (5) cannot be the unbiased estimator of $\delta_{q}^{\prime }$ when $s_{1}\neq s_{2}$, because *g* (2) is not distributed as non-central *t* variate in this situation. Even if $n_{1}$ and $n_{2}$ vary, *g* (2) roughly estimates the same parameter, given the ratio *q* is fixed.

Next, the $e^{\text{biased}}$ (12) is a biased estimator of $\epsilon_{r}$ (11), but $\epsilon_{r}$ (11) equals to the other parameters in the particular situation. When $s_{1}=s_{2}$, $\epsilon_{r}=\delta$, and $e^{\text{biased}}$ (12) equals to *g* (2), and is independent of the sample sizes. When $s_{1}\neq s_{2}$ and $n_{1}=n_{2}$, $\epsilon_{r}=\delta_{q}^{\prime }$. In this case, $e^{\text{biased}}$ (12) equals to *g* (2) and is also independent of the sample sizes. While *d* (5) is a biased estimator of $\delta_{q}^{\prime }$, *e* (15) is its unbiased estimator. Therefore, usage of *e* (15) is always preferable to *d* (5) in this situation. When $s_{1}\neq s_{2}$ and $n_{1}\neq n_{2}$, $e^{\text{biased}}$ (12) depends on the rate $r=n_{1}/n_{2}$. Therefore, strictly speaking, multiple $e^{\text{biased}}$s can be comparable only when the sample size ratio *r* is identical.

The effect size estimator $\overset{ˆ}{\delta }$ (8) did not have a defined parameter, but when $n_{1}=n_{2}$ and $s_{1}=s_{2}$, $\overset{ˆ}{\delta }$ (8) equals to *g* (2) and $e^{\text{biased}}$ (12), and is independent of sample size. Under $n_{1}=n_{2}$ and $s_{1}\neq s_{2}$, $\overset{ˆ}{\delta }$ (8) also equals to *g* (2) and suffers from the same problem as it. Under $n_{1}\neq n_{2}$ and $s_{1}\neq s_{2}$, the value of $\overset{ˆ}{\delta }$ (8) is no longer the same as *g* (2), and precise discussion on its behavior is hinderred by the lack of its parameter definition. When trying to consider $\overset{ˆ}{\delta }$ (8) as a noncentral t-variate like the other effect sizes, its degree of freedom should be about $n_{1}+n_{2}-2$, and $n_{1}$ and $n_{2}$ should affect the degree of freedom under $s_{1}\neq s_{2}$.

Unlike *g* (2) or $e^{\text{biased}}$ (12), $c^{\text{biased}}$ (10) is always independent of the sample size.

The behavior of the unbiased estimator of the effect sizes (*d* (5), *e* (15), and *c* (17)) are almost identical to those that are biased, but they slightly increase as the sample sizes become large. This is because of the correction coefficient *J* (6), and its behavior is illustrated in detail in [14].

In summary, in terms of the effect size of the difference between two means, usage of *e* (15) is preferable to *d* (5) or $\overset{ˆ}{\delta }$ (8), and *e* (15) can be the remedy for application of effect size of the difference under heteroscedasticity. However, when the ratio of the two sample sizes cannot be set as uniform under heteroscedasticity, neither *d* (5) nor *e* (15) can be precisely compared. This is a form of the Behrens-Fisher problem, which cannot be solved strictly.

### 5.3 Potential applications of the new effect sizes

The effect size *e* (15) has a vast applicable range covering all kinds of natural and social sciences. This is because *e* (15) corresponds to Welch's *t* test, whose use is nowadays encouraged over Student's *t* test (e.g., [26]). The effect size *e* (15) is the best option, especially when the ratio of the sample sizes of two groups can be fixed. The effect size *c* (17) has a relatively narrower range regarding the application. In comparison of paired two groups (the difference in pairs vs. 0) and in some simulation studies (result of simulation vs. the optimal value) or physics (result of experiment vs. physical constant), an effect size of the constant may be needed.

## Declarations

### Author contribution statement

Satoshi Aoki: Conceived and designed the analysis; Analyzed and interpreted the data; Contributed analysis tools or data; Wrote the paper.

### Funding statement

This study was partly supported by National Bioresource Project from AMED, grant number 16km0210053j0005.

### Competing interest statement

The authors declare no conflict of interest.

### Additional information

Supplementary content related to this article has been published online at https://CRAN.R-project.org/package=es.dif.

## Appendix A

### A.1 Proofs of unbiasedness and variation of *e*

In summary, this proof is an application of the proof in [14] to the statistic *v* in [11]. Suppose two independently and normally distributed populations $N_{1}(\mu_{1},\sigma_{1}^{2})$ and $N_{2}(\mu_{2},\sigma_{2}^{2})$. Their sample means are ${\overset{¯}{Y}}^{1}$ and ${\overset{¯}{Y}}^{2}$, and their samples are $Y_{i}^{1}$ ($i=1,...,n_{1}$) and $Y_{i}^{2}$ ($i=1,...,n_{2}$). The statistic $e^{\text{biased}}$ (12) between them can be converted into

(18) $$\sqrt{\overset{˜}{n}}e^{\text{biased}}=\frac{({\overset{¯}{Y}}^{1}-{\overset{¯}{Y}}^{2})/\sqrt{\left(\sigma_{1}^{2}/n_{1})+(\sigma_{2}^{2}/n_{2}\right)}}{\sqrt{wf/f}},$$

where

$$w=\frac{s_{1}^{2}/n_{1}+s_{2}^{2}/n_{2}}{\left(\sigma_{1}^{2}/n_{1})+(\sigma_{2}^{2}/n_{2}\right)}.$$

Here, since $N_{1}$ and $N_{2}$ are independently and normally distributed, the numerator of (18) has the normal distribution of N(θ,1), where

$$\theta =\frac{\mu_{1}-\mu_{2}}{\sqrt{\left(\sigma_{1}^{2}/n_{1}+\sigma_{2}^{2}/n_{2}\right)}},$$

and the $s_{i}^{2}$ is the same as (3). In the denominator, *wf* is approximately distributed as $\chi^{2}(f)$ [11]. Therefore, $\sqrt{\overset{˜}{n}}e^{\text{biased}}$ is distributed as a non-central *t* variate with the non-centrality parameter *θ* and approximate degree of freedom *f* (16). From the nature of the non-central *t* distribution [27], the expected value of $e^{\text{biased}}$ (12) is

$$E(\sqrt{\overset{˜}{n}}e^{\text{biased}})=\theta \frac{\sqrt{f/2}\Gamma \{(f-1)/2\}}{\Gamma (f/2)}\;E(e^{\text{biased}})=\theta /\sqrt{\overset{˜}{n}}/J(f).$$

Now, assuming $r=n_{1}/n_{2}$, then $\theta /\sqrt{\overset{˜}{n}}=\epsilon_{r}$. In this case, the expected value of *e* (15) is

$$E(e)=E\{e^{\text{biased}}J(f)\}=E(e^{\text{biased}})J(f)=\{\theta /\sqrt{\overset{˜}{n}}/J(f)\}J(f)=\epsilon_{r}.$$

Thus, *e* (15) is an unbiased estimator of $\epsilon_{r}$ (11). The variation of $e^{bised}$ (12) is

$$\mathrm{var}(\sqrt{\overset{˜}{n}}e^{\text{biased}})=\frac{f}{f-2}(1+\theta^{2})-\theta^{2}/J^{2}(f)\mathrm{var}(e^{\text{biased}})=\frac{f}{f-2}(1/\overset{˜}{n}+\theta^{2}/\overset{˜}{n})-\theta^{2}/J^{2}(f)/\overset{˜}{n}.$$

Therefore, the variation of *e* (15) is

$$\mathrm{var}(e)=\mathrm{var}\{e^{\text{biased}}J(f)\}=\frac{f}{f-2}J^{2}(f)\{1/\overset{˜}{n}+{\left(\theta /\sqrt{\overset{˜}{n}}\right)}^{2}\}-{\left(\theta /\sqrt{\overset{˜}{n}}\right)}^{2}=\frac{f}{f-2}J^{2}(f)(1/\overset{˜}{n}+\epsilon_{r}^{2})-\epsilon_{r}^{2}.\;\text{□}$$

### A.2 Proofs of unbiasedness and variation of *c*

The bias correction and derivation of the variance can be proved in the same way as that of *d* (5). The statistic $c^{\text{biased}}$ (10) can be converted into

(19) $$\sqrt{n-1}c^{\text{biased}}=\frac{\left({\overset{¯}{Y}}^{1}-C\right)}{s/\sqrt{n_{1}-1}},$$

and this (19) is distributed as a non-central *t* variate with non-centrality parameter

$$\frac{\mu -C}{\sigma /\sqrt{n_{1}-1}}$$

and degree of freedom $n_{1}-1$. Therefore, the expected value $c^{\text{biased}}$ (10) is

$$E(\sqrt{n_{1}-1}c^{\text{biased}})=\frac{\mu -C}{\sigma /\sqrt{n_{1}-1}}\frac{\sqrt{(n_{1}-1)/2}\Gamma \{(n_{1}-2)/2\}}{\Gamma ((n_{1}-1)/2)}\;E(c^{\text{biased}})=\frac{\mu -C}{\sigma }\frac{1}{J(n_{1}-1)}\;E(c^{\text{biased}})=\gamma /J(n_{1}-1).$$

Because $c=c^{\text{biased}}J(n_{1}-1)$, the expected value of *c* (17) is

$$E(c)=E\{c^{\text{biased}}J(n_{1}-1)\}=E(c^{\text{biased}})J(n_{1}-1)=\gamma .$$

Thus, *c* is an unbiased estimator of the effect size parameter *γ* (9). The variation of $c^{\text{biased}}$ (10) is

$$\mathrm{var}(\sqrt{n_{1}-1}c^{\text{biased}})=\frac{n_{1}-1}{n_{1}-3}\{1+{\left(\frac{\mu -C}{\sigma /\sqrt{n_{1}-1}}\right)}^{2}\}\;-{\left(\frac{\mu -C}{\sigma /\sqrt{n_{1}-1}}\right)}^{2}\frac{1}{J^{2}(n_{1}-1)}\;\mathrm{var}(c^{\text{biased}})=\frac{n_{1}-1}{n_{1}-3}\{\frac{1}{n_{1}-1}+{\left(\frac{\mu -C}{\sigma }\right)}^{2}\}\;-{\left(\frac{\mu -C}{\sigma }\right)}^{2}\frac{1}{J^{2}(n_{1}-1)}\;\mathrm{var}(c^{\text{biased}})=\frac{n_{1}-1}{n_{1}-3}(\frac{1}{n_{1}-1}+\gamma^{2})-\gamma^{2}\frac{1}{J^{2}(n_{1}-1)}.$$

Therefore, the variation of *c* (17) is

$$\mathrm{var}(c)=\mathrm{var}\{c^{\text{biased}}J(n_{1}-1)\}=\mathrm{var}(c^{\text{biased}})J^{2}(n_{1}-1)=\frac{n_{1}-1}{n_{1}-3}J^{2}(n_{1}-1)(\frac{1}{n_{1}-1}+\gamma^{2})-\gamma^{2}.\;\text{□}$$

### A.3 Proof of consistency of *c*

First, we treat the proof of *c*, which is simpler than that of *e*. For the proof, we introduce a lemma.

Lemma 1 *Assume random samples* $Y_{1}^{1},...,Y_{n}^{1}$ *from the population with the population mean* $\mu_{1}$ *and the population variance* $\sigma_{1}^{2}$*, and consider a parameter β and its statistic* $b=b(Y_{1}^{1},...,Y_{n}^{1})$*. Then,* *[b is an unbiased estimator of β, and* $\lim_{n\to \infty }\mathrm{var}(b)\to 0$*]* ⇒ *[b is a consistent estimator of β.]*

Proof

$$E(|b-\beta {|}^{2})=E{\left(b-\beta \right)}^{2}+\mathrm{var}(b-\beta )={\left\{E(b)-E(\beta )\right\}}^{2}+\mathrm{var}(b)={\left\{E(b)-\beta \right\}}^{2}+\mathrm{var}(b)$$

Given E(b)=β and $\lim_{n\to \infty }\mathrm{var}(b)\to 0$,

$$\lim_{n_{\to }\infty }[{\left\{E(b)-\beta \right\}}^{2}+\mathrm{var}(b)]\to 0.$$

Therefore, *b* is a mean square consistent estimator of *β*, namely,

$$\lim_{n_{\to }\infty }E\{|b-\beta {|}^{2}\}\to 0.$$

Here, for an arbitrary positive number *ε*, by applying Chebyshev's inequality [28], we get

$$P(|b-\beta |\geq \varepsilon )=P(|b-\beta {|}^{2}\geq \varepsilon^{2})\leq E(|b-\beta {|}^{2})/\varepsilon^{2}.$$

From the result shown above, we can say

$$\lim_{n\to \infty }E(|b-\beta {|}^{2})/\varepsilon^{2}\to 0.$$

Therefore, using the squeeze theorem, we get

$$\lim_{n\to \infty }P(|b-\beta |\geq \varepsilon )\to 0.$$

Thus, *b* is a consistent estimator of *β*. □

Now, we move on to the proof of *c* (17). When $n_{1}\to \infty$, the variance of *c* will be

$$\lim_{n_{1}\to \infty }\mathrm{var}(c)=\lim_{n_{1}\to \infty }\frac{n_{1}-1}{n_{1}-3}J^{2}(n_{1}-1)(\frac{1}{n_{1}-1}+\gamma^{2})-\gamma^{2}\to 1\cdot J^{2}(\infty )(\frac{1}{\infty }+\gamma^{2})-\gamma^{2}=0.$$

Thus, $\lim_{n_{1}\to \infty }\mathrm{var}(c)\to 0$, and *c* is an unbiased estimator of *γ*. Therefore, based on Lemma 1, *c* (17) is a consistent estimator of *γ* (9).  □

### A.4 Proof of consistency of *e*

On the other hand, *e* (15) consists of two populations. Therefore, a variation of the previous lemma is necessary.

Lemma 2 *Assume two random samples* $Y_{1}^{1},...,Y_{n_{1}}^{1}$ *and* $Y_{1}^{2},...,Y_{n_{2}}^{2}$ *from the two mother populations with the population means* $\mu_{1}$ *and* $\mu_{2}$*, and the population variance* $\sigma_{1}^{2}$ *and* $\sigma_{2}^{2}$*, respectively. Consider a parameter β and its statistic* $b=b(Y_{1}^{1},...,Y_{n_{1}}^{1};Y_{1}^{2},...,Y_{n_{2}}^{2})$*. Then,* *[b is an unbiased estimator of β, and* $\lim_{\left(n_{1},n_{2})\to (\infty ,\infty \right)}\mathrm{var}(b)\to 0$*]* ⇒ *[b is a consistent estimator of β.]*

This lemma can be proved in the same way as Lemma 1.

Now, consider $n_{1}=r\phi$ and $n_{2}=\phi$ to assume ϕ→∞, which equals to $\left(n_{1},n_{2})\to (\infty ,\infty \right)$. Note that r>0 and θ>0, since $n_{1}\geq 1$ and $n_{2}\geq 1$. Using *r* and *ϕ*, *f* (6) and $\overset{˜}{n}$ (14) can be expressed as

$$f=\frac{{\left(s_{1}^{2}/r+s_{2}^{2}\right)}^{2}}{s_{1}^{4}/\{r^{2}(r\phi -1)\}+s_{2}^{4}/\{{\left(1/r\right)}^{2}(\phi -1)\}}$$

and

$$\overset{˜}{n}=\frac{r\phi }{r+1}.$$

Therefore, when ϕ→∞, the variance of *e* (15) will be

$$\lim_{\phi \to \infty }\mathrm{var}(e)=\lim_{\phi \to \infty }\frac{f}{f-2}J^{2}(f)(\frac{1}{\overset{˜}{n}}+\epsilon_{r}^{2})-\epsilon_{r}^{2}=\lim_{\phi \to \infty }\frac{1}{1-2/f}J^{2}(f)(\frac{1}{\overset{˜}{n}}+\epsilon_{r}^{2})-\epsilon_{r}^{2}\to \frac{1}{1-2/\infty }J^{2}(\infty )(\frac{1}{\infty }+\epsilon_{r}^{2})-\epsilon_{r}^{2}=\frac{1}{1-0}\cdot 1\cdot (0+\epsilon_{r}^{2})-\epsilon_{r}^{2}=0.$$

The limit does not contain *r*, meaning $\lim_{\left(n_{1},n_{2})\to (\infty ,\infty \right)}\mathrm{var}(e)$ always gives an identical value 0. Also, *e* is an unbiased estimator of $\epsilon_{r}$ (11). Therefore, based on Lemma 2, *e* (15) is a consistent estimator of $\epsilon_{r}$ (11).  □

### A.5 Simulation source code

The simulation study in this article was conducted using the following code in R:

library(es.dif)  library(Matrix)  library(metafor)    rep<-100000  n_sd<-10  sampleSize<-matrix(0,nrow=9,ncol=2)  sampleSize[1,]<-c(10,10)  sampleSize[2,]<-c(10,20)  sampleSize[3,]<-c(10,30)  sampleSize[4,]<-c(20,10)  sampleSize[5,]<-c(20,20)  sampleSize[6,]<-c(20,30)  sampleSize[7,]<-c(30,10)  sampleSize[8,]<-c(30,20)  sampleSize[9,]<-c(30,30)    d<-numeric(rep)  var_d<-numeric(rep)  ci_lb_d<-numeric(rep)  ci_ub_d<-numeric(rep)    e<-numeric(rep)  var_e<-numeric(rep)  ci_lb_e<-numeric(rep)  ci_ub_e<-numeric(rep)    Bonett<-numeric(rep)  var_Bonett<-numeric(rep)  ci_lb_Bonett<-numeric(rep)  ci_ub_Bonett<-numeric(rep)    result_d<-matrix(0,nrow=(n_sd)*nrow(sampleSize),   ncol=4)  result_e<-matrix(0,nrow=(n_sd)*nrow(sampleSize),   ncol=4)  result_Bonett<-matrix(0,nrow=(n_sd)*nrow(sampleSize),   ncol=4)  counter <-1  for(k in 1:nrow(sampleSize))  {   for(j in 1:n_sd)   {   for(i in 1:rep)   {   data1<-rnorm(sampleSize[k,1],1,1)   data2<-rnorm(sampleSize[k,2],0,j)     temp_d <-es.d(data1,data2,   vector_out=T)   d[i] <- temp_d[1]   var_d[i]<- temp_d[2]   ci_lb_d[i]<-temp_d[3]   ci_ub_d[i]<-temp_d[4]     temp_e <-es.e(data1,data2,   vector_out=T)   e[i] <- temp_e[1]   var_e[i]<- temp_e[2]   ci_lb_e[i]<-temp_e[3]   ci_ub_e[i]<-temp_e[4]   temp_Bonett   <-summary(escalc(measure="SMDH",  m1i=mean(data1),m2i=mean(data2),  sd1i=sd(data1),sd2i=sd(data2),n1i=length(data1),   n2i=length(data2)))   Bonett[i] <- temp_Bonett[1,1]   var_Bonett[i]<- temp_Bonett[1,2]   ci_lb_Bonett[i]<-temp_Bonett[1,5]   ci_ub_Bonett[i]<-temp_Bonett[1,6]   }   result_d[counter,]<-c(mean(d),  mean(var_d), mean(ci_lb_d),mean(ci_ub_d))   result_e[counter,]<-c(mean(e),  mean(var_e), mean(ci_lb_e),mean(ci_ub_e))   result_Bonett[counter,]   <- c(mean(Bonett),  mean(var_Bonett), mean(ci_lb_Bonett),  mean(ci_ub_Bonett))   counter <- counter + 1   }  }
